# Early spectral EEG in preterm infants correlates with neurocognitive outcomes in late childhood

**DOI:** 10.1038/s41390-021-01915-7

**Published:** 2022-01-10

**Authors:** Tone Nordvik, Eva M. Schumacher, Pål G. Larsson, Are H. Pripp, Gro C. Løhaugen, Tom Stiris

**Affiliations:** 1 Institute of Clinical Medicine, Faculty of Medicine, University of Oslo, Oslo, Norway; 2 Department of Neonatal Intensive Care, Oslo University Hospital, Ullevål, Oslo, Norway; 3 Department of Neurosurgery, Oslo University Hospital, Oslo, Norway; 4 Oslo Center of Biostatistics and Epidemiology, Research Support Services, Oslo University Hospital, Oslo, Norway; 5 Department of Pediatrics, Sørlandet Hospital, Arendal, Norway

## Abstract

**Background:**

Evidence regarding the predictive value of early amplitude-integrated electroencephalography (aEEG)/EEG on neurodevelopmental outcomes at school age and beyond is lacking. We  aimed to investigate whether there is an association between early postnatal EEG and neurocognitive outcomes in late childhood.

**Methods:**

This study is an observational prospective cohort study of premature infants with a gestational age <28 weeks. The total absolute band powers (tABP) of the delta, theta, alpha, and beta bands were analyzed from EEG recordings during the first three days of life. At 10–12 years of age, neurocognitive outcomes were assessed using the Wechsler Intelligence Scale for Children 4th edition (WISC-IV), Vineland adaptive behavior scales 2nd edition, and Behavior Rating Inventory of Executive Function (BRIEF). The mean differences in tABP were assessed for individuals with normal versus unfavorable neurocognitive scores.

**Results:**

Twenty-two infants were included. tABP values in all four frequency bands were significantly lower in infants with unfavorable results in the main composite scores (full intelligence quotient, adaptive behavior composite score, and global executive composite score) on all three tests (*p* < 0.05).

**Conclusions:**

Early postnatal EEG has the potential to assist in predicting cognitive outcomes at 10–12 years of age in extremely premature infants <28 weeks’ gestation.

**Impact:**

- Evidence regarding the value of early postnatal EEG in long-term prognostication in preterm infants is limited.
- Our study suggests that early EEG spectral analysis correlates with neurocognitive outcomes in late childhood in extremely preterm infants.
- Early identification of infants at-risk of later impairment is important to initiate early and targeted follow-up and intervention.

## Introduction

Despite recent advances in the care of premature infants, morbidity remains high.^1,2^ Infants born extremely premature are at-risk of neurodevelopmental impairments.^1,2^ Detection of structural damage and compromised brain function is important, as early recognition of at-risk infants is necessary to implement appropriate care and follow-up.^3^ However, predicting impairment is challenging.^4,5^ Current available biomarkers, including cranial ultrasonography (CUS) and magnetic resonance imaging (MRI) have limitations.^6,7^ Whereas both CUS and MRI have relatively high specificity and negative predictive value in predicting outcomes, the sensitivity, and positive predictive value are poor.^6,7^ Many children with adverse neuroimaging may have normal neurodevelopment, and some children with normal neuroimaging may develop severe impairments. There is a need for supplemental monitoring tools to help identify infants at-risk.

With the development of the quantitative analysis of electroencephalography (EEG), EEG is becoming more accessible for clinical use in the neonatal intensive care unit. It is now routinely used in, for example, term infants with hypoxic–ischemic encephalopathy, where a persistently abnormal EEG at 48 h of age is associated with poor outcomes.^8–10^ The use in preterm infants is promising but less clear.^11–14^ Although there are several studies suggesting a predictive value of early EEG, the evidence was more equivocal when the data were combined.^15,16^ A recent meta-analysis by Fogtmann et al. claims that amplitude-integrated EEG (aEEG)/EEG may have potential as a predictor of later neurodevelopmental outcomes.^15^ Focusing on cognitive outcomes, a systematic review by Kong et al. found preliminary evidence that background EEG features can predict cognitive outcomes in very preterm infants.^16^ Both studies, however, concluded that more studies are needed to draw definite conclusions, emphasizing the wide heterogeneity in both EEG metrics and outcome measures used, which complicated the meta-analysis of the data.

The majority of studies assessing the use of aEEG/EEG in prognostication have a relatively brief follow-up of 18–36 months.^15^ Cognitive and neuropsychological dysfunction, which is the most common sequelae after preterm birth, may emerge later, and some deficits become evident only when cognitive demands increase at school age. More subtle cognitive disabilities may therefore be missed on early assessment.^17^ In the EXPRESS study, less than half of the children with moderate/severe disabilities at 6.5 years of age were correctly identified at 2.5 years.^1^ Few studies, however, assess the value of early EEG in predicting outcomes in later childhood, and the association of early EEG with neurodevelopmental outcomes at school age and beyond has yet to be explored.

We have previously reported a significant association between total absolute band power (tABP) monitored the first 3 days of life and developmental outcomes at 24 months corrected age in extremely premature children <28 weeks’ gestation.^18^ We hypothesize that tABP over the first three days of life in extremely premature infants may help predict neurocognitive outcomes in late childhood. Thus, the aim of the study is to examine whether the associations between tABP and neurodevelopmental outcomes persist into late childhood, with a focus on cognitive and neuropsychological impairment.

## Methods

The study is an observational prospective cohort study conducted at Oslo University Hospital, Ullevaal, Norway. The study was approved by the Regional Committee for Medical Ethics (REK 2011/1214) and the Norwegian Data Protection Supervisor. We refer to previously published papers for more details regarding the study population and EEG technique.^19,20^

### Subjects

**Fig. 1:**
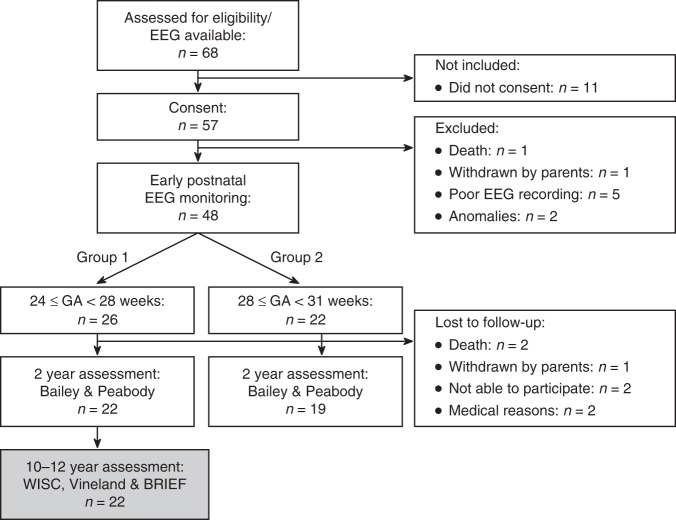
Flow chart of the participants. 22 children from group 1 were included in this study. WISC Wechsler Intelligence Scale for Children. BRIEF Behavioral Rating Inventory of Executive Function.

Forty-eight infants with gestational age (GA) < 31 weeks were included in the original study over a 21-months period from 2004 to 2006 after parental consent was obtained. EEG recording was started within 12 h after birth and continued until the infants reached 72 h of life. The infants were divided into two groups based on GA: group one from GA 24 + 0–27 + 6 and group two from GA 28 + 0–30 + 6. At 2 years of age, 41 infants were seen for follow-up: 22 from group one and 19 from group two. As a significant association between early multichannel EEG and outcomes was found only in group one,^18^ 22 infants with GA < 28 weeks were included in this part of the study (Fig. 1).

Clinical characteristics and type of sedation were prospectively recorded. The children were scored using a total disease burden score based on the presence of severe ROP, BPD, and brain injury (Supplementary Material Table 1).^21,22^ Socioeconomic status was assessed using the Hollingshead four-factor scale.^23^

### Measurements

#### EEG

EEG was recorded with the NicoletOne™ version 5.2 EEG system (Natus, Calif.)

Eight electrodes were placed according to a modified version of the international 10–20 system using the following channels: Fp1, Fp2, P3, P4, T3, T4, O1, and O2. Bipolar recordings from eight channels were analyzed. The NicoletOne EEG review software trend analysis was used to calculate ABP during the first 3 days of life. Absolute band power (ABP), defined as the integral of all the power values within a frequency range and expressed in µV^2^, was calculated and used as a measurement of overall brain activity. ABP was first calculated for each consecutive second for the four frequency bands delta, theta, alpha and beta (*δ*, 1.0–4.0; *θ*, 4.0–8.0; *α*, 8.0–13.0; *β*, 13.0–30.0 Hz). tABP was then calculated for each band as the sum of the band’s ABP over all eight channels. Finally, the median tABP values for days 1, 2, and 3 were calculated for each frequency band in each infant.^18,19^ To remove artefacts automated trimming with the removal of 5% of the highest tABP values in each patient was performed, as this has previously been reported to show the closest relationship to the visual edited method.^19^ Due to a small skewness in the median tABP values, a log transformation was done.

#### Clinical assessment

At 10–12 years of age, the children were assessed by a clinical neuropsychologist and a pediatric neurologist. The children were subjected to three tests that assessed different aspects of their cognitive abilities, in addition to a general neurological examination.

General intelligence was tested using the Wechsler Intelligence Scale for Children, 4th edition (WISC-IV). The WISC-IV consists of 10 subtests in four domains (verbal comprehension, perceptual reasoning, working memory, and processing speed), which form the composite score full-scale intelligence quotient (FSIQ).^24^ The FSIQ is considered the most representative measurement of global intellectual functioning.^25^ Age-appropriate standardized Scandinavian norms were applied to calculate IQ scores. A score more than 1 SD below the mean (mean: 100; SD: 15) was defined as unfavorable. Children who were not able to perform all the subtests due to severe intellectual impairment or autism were coded as having severe cognitive impairment.

The Vineland Adaptive Behavior Scales-II was used to assess the children’s adaptive skills. This parent-reported survey focuses on three domains of adaptive behavior: communication, daily living skills and social functioning. The three domains generate an adaptive behavior composite. As with the WISC-IV, a score more than 1 SD below the mean (mean: 100; SD: 15) was considered unfavorable.^26^

The parental form of the Behavioral Rating Inventory of Executive Function (BRIEF) was used to assess behavioral problems and executive functions in everyday life. The BRIEF consists of 86 items across 8 clinical scales and two validity scales. For this study, the overall composite score (GEC) of the test was used as the main measure of executive functioning, and scores ≥65 were considered unfavorable.^27^

#### Data analysis

Continuous data were assessed by *t*-tests and presented as the mean ± SD. Categorical variables were assessed by chi-square or Fisher’s exact tests and presented as proportions (percentages). Ordinal data were assessed by Mann–Whitney *U* test and presented as either median with quartiles or number with percentages.

The mean differences in tABP were assessed for individuals with normal versus unfavorable neurocognitive outcomes using linear mixed models for repeated measurements with subject-specific intercepts. We adjusted for the potential confounding factors GA, small for gestational age (SGA), sex, and socioeconomic status (SES). A regression analysis was performed to assess which day was the optimal time point for EEG registration in assessing long-term prognosis based on R-squared values. A *p*-value of <0.05 was considered statistically significant.

The data were analyzed using Stata/SE 15.0 (StataCorp LLC, College Station, TX).

## Results

### Patients

All 22 patients with GA < 28 weeks assessed at 2 years corrected age were assessed at 10–12 years of age. None were lost to follow-up between 2 and 10–12 years of age.

The demographic and clinical data of the infants stratified according to outcomes are summarized in Table 1. The mean GA was 26.4 ± 1.0 weeks (mean±SD), and the mean birth weight (BW) was 858.9 g ± 188.7 g (mean± SD).

**Table 1:** Perinatal characteristics of the study infants stratified according to the outcome.

Perinatal charcteristics	WISC >85	WISC ≤ 85	Vineland > 85	Vineland ≤ 85	BRIEF < 65	BRIEF ≥ 65
	*n* = 17	*n* = 5	*n* = 15	*n* = 7	*n* = 17	*n* = 5
GA (wk), mean ± SD	26.6 ± 0.9	25.7 ± 1.4	26.5 ± 0.9	26.1 ± 1.4	26.6 ± 0.9	25.7 ± 1.4
BW (g), mean ± SD	897 ± 191	729 ± 118	852 ± 150	872 ± 269	897 ± 191	729 ± 118
Male sex, *n* (%)	10/17 (59)	3/5 (60)	8/15 (53)	5/7 (71)	10/17 (59)	3/5 (60)
SGA, *n* (%)	5/17 (29)	1/5 (20)	5/15 (33)	1/7 (14)	5/17 (29)	1/5 (20)
SES, *n* (%)						
1	0 (0)	2 (40)	0 (0)	2 (29)*	0 (0)	2 (40)
2	3 (18)	0 (0)	2 (13)	1 (14)	3 (18)	0 (0)
3	7 (41)	3 (60)	6 (40)	4 (57)	7 (41)	3 (60)
4	7 (41)	0 (0)	7 (47)	0 (0)	7 (41)	0 (0)
Apgar 5 min, median IQR	7 (6, 8)	5 (5, 9)	7 (6, 8)	7 (5, 9)	7 (6, 8)	5 (5, 9)
Surfactant, *n* (%)	14/17 (82)	5/5 (100)	12/15 (80)	7/7 (100)	14/17 (82)	5/5 (100)
Sedation, *n* (%)	4/17 (24)	1/5 (20)	3/15 (20)	2/7 (29)	4/17 (24)	1/5 (20)
Early sepsis, *n* (%)	1/17 (6)	2/5 (40)	1/15 (7)	2/7 (29)	1/17 (6)	2/5 (40)
Late sepsis, *n* (%)	2/17 (12)	2/5 (40)	2/15 (13)	2/7 (29)	2/17 (12)	2/5 (40)
TDB, *n* (%)						
0	14 (82)	2 (40)*	12 (80)	4 (57)	14 (82)	2 (40)*
1	2 (12)	1 (20)	2 (13)	1 (14)	2 (12)	1 (20)
2	1 (6)	1 (20)	1 (7)	1 (14)	1 (6)	1 (20)
3	0 (0)	1 (20)	0 (0)	1 (14)	0 (0)	1 (20)
NEC (%)	2/5 (40)	0/17 (0)*	2/7 (29)	0/15 (0)	2/5 (40)	0/17 (0)*
PDA (%)	4/5 (80)	2/17 (12)*	4/7 (57)	2/15 (13)	4/5 (80)	2/17 (12)*

Data are presented as mean ± standard deviation (SD), number (%) or median with interquartile range (IQR).

*WISC* Wechsler Intelligence Scale for Children, *BRIEF* Behavioral Rating Inventory of Executive Function, *GA* gestational age, *BW* birth weight, *SGA* small for gestational age, *SES* socioeconomic status, *TDB* total disease burden, *NEC* necrotizing enterocolitis, *PDA* patent ductus arteriosus.

*p < 0.05.

Thirteen were male (59%) and 5 were multiples (23%). Six infants were SGA (27%), defined as a weight below the 10th percentile. The incidence of intraventricular hemorrhage (IVH) ≥3 detected on cerebral ultrasound was 1 (5%). Three infants had early sepsis (14%), 2 developed necrotizing enterocolitis (NEC) (9%), 6 infants had medically treated patent ductus arteriosus (PDA) (27%), and 6 infants developed bronchopulmonary dysplasia (BPD) (27%). Nine of the infants received inotropic support during the EEG recording. None of the infants received continuous sedation, but 5 infants were given a morphine bolus (0.1–0.2 mg/kg) for intubation.

### Neurodevelopmental outcomes

At the 10–12-year assessment (mean age: 11,5 ± 0,6), two children (9%) had a diagnosis of cerebral palsy, and one child (4.5%) had autism spectrum disorder.

The mean WISC FSIQ was 88.82; the Vineland GAF was 89.2; and the BRIEF GEC, 51.5. A total of 5/22 children (23%) had unfavorable scores on the WISC FSIQ and the composite score of the BRIEF test, whereas 7/22 (32%) had unfavorable scores on the adaptive behavior composite of the Vineland test (Table 2). The children with the unfavorable score on the WISC test were the same children who had the unfavorable score on the BRIEF test.

**Table 2:** Developmental outcomes at 10–12 years of age.

	WISC-IV	Vineland-II	BRIEF
*n*	22	22	22
Normal outcome	17 (77%)	15 (68%)	17 (77%)
Unfavorable outcome	5 (23%)	7 (32%)	5 (23%)

Data are presented as number (%).

*WISC-IV* Wechsler Intelligence Scale for Children 4th edition, *Vineland-II* Vineland Adaptive Behavior Scales 2nd edition, *BRIEF* Behavioral Rating Inventory of Executive Function.

### Relationship between tABP and neurodevelopmental outcomes

**Fig. 2:**
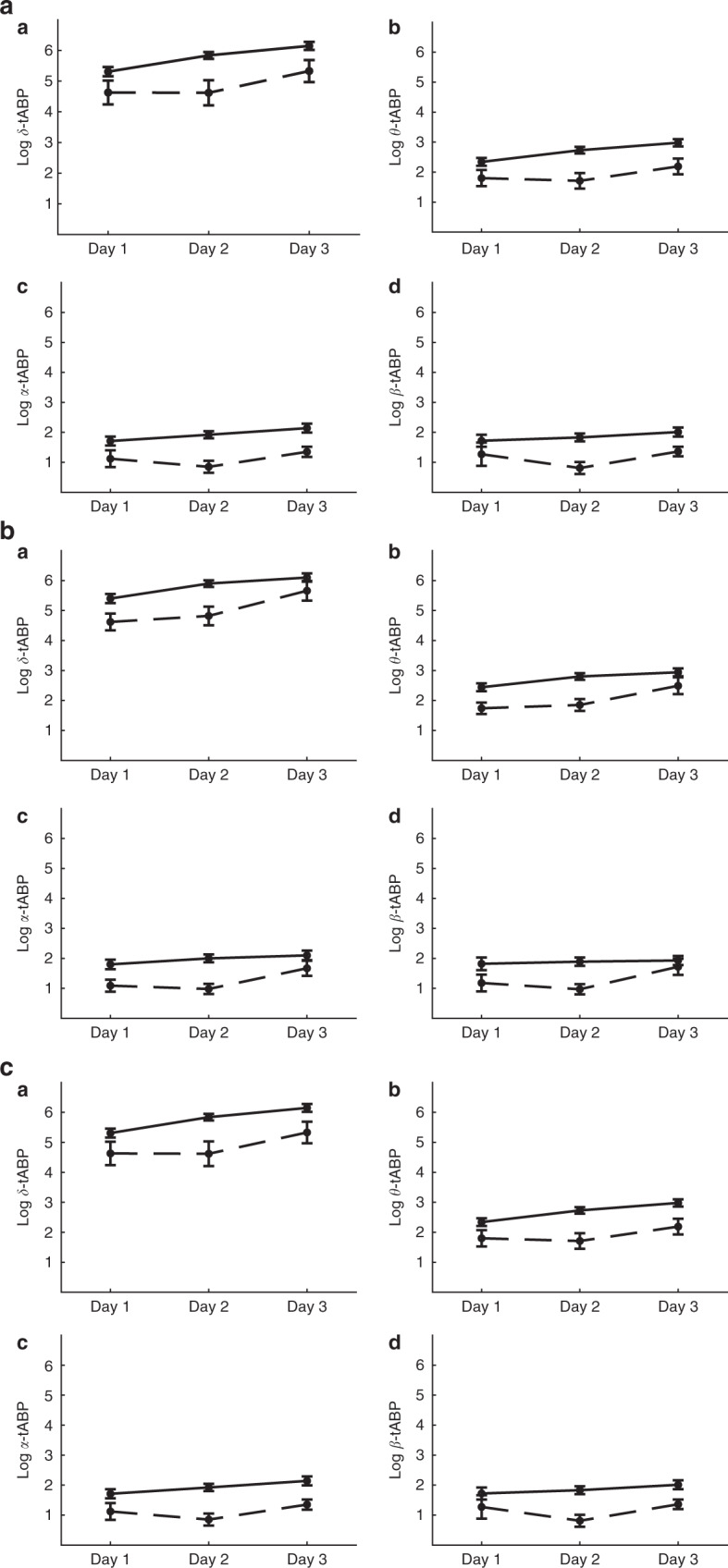
tABP over first three days of life for normal and unfavorable neurocognitive outcomes. The figures show the results for the WISC (**a**), Vineland (**b**), and BRIEF tests (**c**). Panels a–d show the log-transformed tABP values for the four different frequency bands on days 1, 2, and 3. The results are presented as the mean of the medians ± SEM. The solid line represents the children with normal scores on the developmental tests, whereas the dotted line represents the children with unfavorable scores. The tABP values were significantly lower for the infants with unfavorable scores than for the infants with normal scores for all four frequency bands and for all three tests, with a *p*-value < 0.05.

There were significant associations between tABP values measured during days 1, 2, and 3 and cognitive outcomes at 10–12 years of age. This was seen for all four frequency bands and for all three functional outcomes. The children with unfavorable outcomes at 10–12 years of age had significantly lower tABP during days 1, 2, and 3 compared to the children with normal scores on the cognitive tests (*p*-value < 0.05) (Fig. 2a–c). The best explanatory ability based on *R*-squared values was found on day 2 of life (Table 3). After adjusting for GA, SGA, BW, and SES, the results remained significant for all three tests for the delta and theta bands (Table 4).

**Table 3:** Overview of adjusted *R*^2^-value for each test and days 1, 2, and 3.

	Delta	Theta	Alpha	Beta
WISC				
Day 1	0.123	0.113	0.103	0.005
Day 2	0.435^a^	0.448^a^	0.445^a^	0.399^a^
Day 3	0.228	0.270	0.235	0.166
Vineland				
Day 1	0.237	0.288	0.221	0.092
Day 2	0.421^a^	0.483^a^	0.499^a^	0.406^a^
Day 3	0.050	0.079	0.055	−0.021
BRIEF				
Day 1	0.123	0.113	0.103	0.005
Day 2	0.435^a^	0.448^a^	0.445^a^	0.399^a^
Day 3	0.228	0.270	0.235	0.166

^a^Day with the highest *R*^2^-value.

**Table 4:** Mean difference in the log-transformed tABP value for the different frequency bands between normal and unfavorable test score for unadjusted and adjusted values (adjusted for gender, GA, SGA, and SES) for day 2 of life.

	Mean difference Unadjusted values	*p*-value	Mean difference Adjusted values	*p*-value
WISC FSIQ				
Logdelta	−0.90 [−1.37,−0.44]	<0.000	−0.71 [−1.27,−0.15]	0.013
Logtheta	−0.78 [−1.18,−0.39]	<0.000	−0.48 [−0.93,−0.03]	0.037
Logalpha	−0.82 [−1.27,−0.36]	<0.000	−0.48 [−0.99, 0.03]	0.067
Logbeta	−0.71 [−1.18,−0.23]	0.004	−0.47 [−1.03, 0.10]	0.109
Vineland				
Logdelta	−0.77 [−1.20,−0.33]	0.001	−0.58 [−1.06,−0.09]	0.019
Logtheta	−0.70 [−1.06,−0.34]	<0.000	−0.45 [−0.82,−0.07]	0.019
Logalpha	−0.72 [−1.13,−0.30]	0.001	−0.50 [−0.92,−0.08]	0.019
Logbeta	−0.59 [−1.03,−0.15]	0.009	−0.49 [−1.00, 0.03]	0.039
BRIEF				
Logdelta	−0.90 [−1.37,−0.44]	<0.000	−0.71 [−1.26,−0.15]	0.013
Logtheta	−0.78 [−1.18,−0.39]	<0.000	−0.48 [−0.93,−0.03]	0.037
Logalpha	−0.82 [−1.27,−0.36]	<0.000	−0.48 [−0.99, 0.03]	0.067
Logbeta	−0.71 [−1.18, 0.23]	0.004	−0.47 [−1.03, 0.10]	0.109

Data are presented as the mean difference with 95% CI.

## Discussion

This study demonstrated a relationship between tABP during the first 3 days of life and neurocognitive outcomes at 10–12 years of age. These results are in accordance with a recently published review.^16^ To our knowledge, this is the first study to show a significant association between early postnatal EEG and cognitive outcomes in late childhood in infants born extremely premature. Furthermore, the strongest association between early EEG and outcomes was seen on day 2 of life.

Several studies have described the changes occurring in spectral powers in the first few days after birth in preterm infants.^19,28–30^ However, little evidence exists regarding the role of early postnatal spectral power analysis in predicting later outcomes for preterm children.^16^ Spectral analysis of the EEG in preterm infants performed close-to-term and in term infants suggested that it can be a useful tool for predicting later outcomes, in particular, neurocognitive outcomes.^31–35^ Cognitive challenges are the most common complications after premature birth, affecting up to 40% of extremely preterm infants.^5,36,37^

In preterm infants, spectral analysis of EEG performed at 35 weeks postmenstrual age (PMA) has been demonstrated to correlate with outcomes at 1 year of age^31^ and at 6 years of age.^32^ Despite examining a more heterogeneous group of premature infants with GA 23–35 (mean GA 29 + 5), Cainelli et al. found a significant association between reduced power in the alpha and beta bands and visual and auditory tasks.^32^ We have previously shown that early postnatal spectral analysis was associated with neurological outcomes at 2 years of age.^18^ Focusing on neurocognitive outcomes, we now found a significant association between reductions in power in the delta, theta, alpha, and beta frequency bands and neurocognitive outcomes in late childhood. With the development of a more automatic assessment of EEG and new analytic tools, spectral analysis is becoming a promising and easy method of analyzing multichannel EEG of preterm infants.^38^

Overall, there is a lack of data on early EEG and its value in predicting outcomes in late childhood. Middel et al. and Feldmann et al.^11,13^ assessing the value of aEEG on outcomes in early school age, concluded that there was no or only limited value of early postnatal aEEG in predicting neuropsychological/cognitive outcomes. There are, however, some important differences between these studies and our study. Both Middel et al. and Feldmann et al. used aEEG in their studies. We used multichannel EEG, enabling an analysis of spectral power. The opposing results may imply that spectral power analysis of multichannel EEG is a better tool than aEEG for predicting neurological outcomes at school age. Cognitive function was also measured at an earlier time point (mean age 5.72 and 7.39) in the studies by Middel et al. and Feldmann et al. Cognitive dysfunction may emerge over time, and some problems, in particular problems with attention and processing speed, may appear with increasing age.^39–41^ Last, they examined a more mature group of preterm infants; Middel et al. included infants with GA 26.0–32.9, and Feldmann et al. included infants with GA < 32 weeks. In our previous study assessing the value of EEG on outcomes at 2 years of age, we were able to find a significant association between early EEG and outcomes only in the most immature infants with GA < 28 weeks of age.^18^ This is in accordance with findings by Klebermass et al., who found higher sensitivities between EEG and outcomes in more immature infants.^42^ The reason for this is not known. One hypothesis is that the most immature brains, being at an earlier stage of ontogenesis, are more susceptible to the exogenous metabolic and physiological stress that they are exposed to after preterm birth.^43^

Accumulating research into the brain connectome has linked alterations in brain networks to problems with behavior and cognition in premature infants.^44^ It has been postulated that preterm birth actually represents a disease of connectivity.^45^ The 2nd trimester is a very important and vulnerable window for connectome development and organization.^46^ At the end of the 2nd trimester, a rich club of interconnected cortical hubs has been established,^47,48^ and the brain’s structural network is already highly efficient. During the 3rd trimester, the principal development involves connections between the core hubs and other parts of the brain.^47^ Prematurity can cause alterations in brain connectivity.^49^ Pandit et al. found widespread reductions in connection strength in tracts involving all cortical lobes and several subcortical structures with increasing prematurity.^49^ It is plausible that preterm birth during the period with extensive development of structural networks will affect EEG and later outcomes more than preterm birth when the functional networks are more established. However, there is much that has yet to be explored regarding the associations between connectivity, outcomes, and the impact of prematurity.

The optimal timing for EEG registration to predict prognosis is unclear.^50,51^ Over the first 3 days of life, there are rapid developmental changes in cortical activity in premature infants.^18,28,30,52^ Several other studies have assessed the optimal time point for predicting neurodevelopmental outcomes in early childhood using aEEG, with conflicting conclusions. Wikstrom et al. found that measurements of interburst percentage and burst suppression at 24–48 h of life were the best predictors of outcomes at 2 years corrected age.^12^ Richardson et al. showed that depressed aEEG background and absence of cycling on day 3 of life were associated with death or poor long-term outcomes at 2 years of age in premature infants with GA < 28 weeks.^50^ Ralser et al., on the other hand, concluded that the optimal time point for predicting adverse neurodevelopmental outcomes at one year of age in infants born before 32 weeks of life is within the first 2 days of life. The highest predictive value was found at 18–24 h, with an additional time period offering a respectable prediction at 30–36 h.^51^ Our study suggested that the second day of life is most informative. As premature babies are subjected to many procedures during the first day of life, being able to delay EEG recording until the 2nd day of life is very desirable.

It is expected that infants with lower tABP and worse neurocognitive outcomes were more ill during the neonatal period. To test for this we scored the children using a total burden of disease score based on data from i.e., Smith et al., who found an association between a count of the three morbidities brain injury, ROP and BPD, and poor outcome defined as FSIQ ≤ 70 (Supplementary Material).^21,22^ Contrary to Smith et al., we defined a score of ≤85 as unfavorable on the neurocognitive tests, as the borderline score may also have functional consequences that are important to identify early.^53,54^ Although the results should be interpreted with care due to the small sample size, the infants with low scores on the cognitive tests in our cohort tend to have a higher total disease burden score and higher prevalence of other neonatal morbidities.

Additional analyses were also performed to account for the major confounding factors known to influence EEG and/or neurocognitive outcomes: GA, BW, SGA, and SES. Feldmann et al. initially found an association between early EEG and neuropsychological outcomes, but this association disappeared when accounting for confounding factors.^13^ The associations between tABP and outcomes remained significant for the delta and theta bands with all three tests when accounting for GA, BW, SGA, and SES and for the alpha and beta bands with the Vineland test. Although the children with normal scores on the WISC test and the BRIEF assessment still had higher spectral powers in the alpha and beta bands after accounting for confounding factors, the difference was no longer statistically significant. As EEG in early life in extremely premature infants is predominated by powers in the lower frequency bands, it was not unanticipated that the delta and theta bands would be most affected in early life.^19,28,29^

We measured SES using the Hollingshead four-factor scale at the 10–12-year follow-up. The use of this tool may be debated. Hollingshead SES was introduced in 1975. It has limitations, particularly in families with one wage earner who is a female.^55^ More recent studies show that maternal education is in fact the most informative measure of SES.^56–58^ Joseph et al. confirmed that socioeconomic disadvantage at birth, indexed by maternal education, was associated with significantly poorer neurocognitive and academic performance at 10 years of age.^59^ As SES can change between birth and 10–12 years of age, measuring SES at an earlier stage might have been more accurate.^59^ Over the first years of life, there is substantial growth in brain tissue and an increase in the number of synapses. The neonatal brain is very sensitive to environmental exposure at this time, and it has therefore been speculated that SES is of most importance in early childhood.^56^ Unfortunately, the data protection at our hospital did not allow the collection of SES data at the two-year follow-up. The data protection regulations have been updated, allowing the measurement of SES in clinical studies.

Another known factor known to affect EEG is the use of morphine. Even a single bolus, i.e., given in association with intubation, is known to depress the EEG for up to 24 h.^60–62^ It is not known how morphine affects tABP. No infants received continuous infusions of morphine, but five neonates received morphine in association with intubation in our cohort (0.15 mg/kg); 4 of these infants had an FSIQ within the normal range; only 1 had a pathological score (20 vs 23.5%, respectively). There was no significant difference in morphine exposure between the normal and pathological groups. We, therefore, find it unlikely that morphine exposure significantly affected our results.

We have previously published data from this cohort on outcomes at 2 years corrected age and the association with tABP.^18^ At 2 years corrected age there were 6 children with score <1 SD below mean on the Bailey-II Mental Developmental Index (MDI). 4 of these (67%) had unfavorable outcomes on all three neurocognitive tests at 10–12 years of age, 1 had the unfavorable score on the Vineland test only, whereas 1 child had a normal score on all three cognitive tests in late childhood. 4/5 (80%) of children with the unfavorable score on all neurocognitive test and 5/7 (71%) with the unfavorable score on the Vineland test only had an unfavorable outcome on the Bailey MDI at 2 years of age. An intriguing question is whether EEG can help distinguish those children who appear to have normal neurocognitive outcomes at early assessment but then later develop impairments. Unfortunately, our sample size is too small to answer this in detail. However, we did observe that the children with normal Bailey MDI but unfavorable outcomes in late childhood had tABP in the lower ranges.

There are several limitations to our study. The main limitation of the present study is the small sample size. We have only two EEG devices in our unit, and not all potential participants could be included due to the lack of devices. For the same reason, multiple pregnancies were often not included, resulting in possible selection bias. We have no data on the perinatal baseline characteristics of the non-recruited infants. Infants who died were excluded. Retrospectively, these should have been considered adverse outcomes (or reported as secondary outcomes). The clinical characteristics of the two infants who died have not been registered. However, the median log tABP values for the two infants are in the lower ranges for all four frequency bands and for all 3 days, supporting our findings. Preferably more advanced and comprehensive neurocognitive assessment tools should have been used but this was unfortunately not feasible due to limited funding and resources. In our study, SES was first measured at late childhood and not at birth or the 2-year follow-up.

The strengths of the study include its prospective design and long, continuous registration with multichannel EEG for up to 72 h. There was no loss to follow-up between 2 years of age and 10–12 years of age. All children were also assessed by the same neuropsychologist, who was blinded to the EEG data.

In conclusion, our data demonstrate that tABP over the first 3 days of life in premature infants with GA < 28 weeks is associated with neurocognitive outcomes in late childhood. For extremely premature infants, early spectral analysis has the potential to assist in the prognostication of long-term neurocognitive outcomes using the WISC-IV, Vineland, and BRIEF tests. This may enable earlier identification of at-risk infants, and EEG can be a useful tool in implementing more targeted and specific follow-up and interventions for this vulnerable group of patients.

## Supplementary information

Supplementary Table 1
