# Supplementary material for: Early spectral EEG in preterm infants correlates with neurocognitive outcomes in late childhood
Source: Pediatr Res. 2022 Jan 10;92(4):1132–9. doi: 10.1038/s41390-021-01915-7 (PMC9586859; doi:10.1038/s41390-021-01915-7)
Supplement: Supplementary file 1 — Supplementary Table 1 [file 41390_2021_1915_MOESM1_ESM.docx]

Table 5: Total Disease Burden

|  | Yes | No |
| --- | --- | --- |
| Brain Injury ^a^ | 1 | 0 |
| BPD ^b^ | 1 | 0 |
| ROP ^c^ | 1 | 0 |

^a^ IVH papile grade ≥3 or PVL ^b^ oxygen requirement at 36 weeks PMA ^c.^ ROP grade ≥3

Maximum score of 3. BPD bronchopulmonary dysplasia. ROP retinopathy of prematurity.
